# Global spectrum of *USH2A* mutation in inherited retinal dystrophies: Prompt message for development of base editing therapy

**DOI:** 10.3389/fnagi.2022.948279

**Published:** 2022-08-10

**Authors:** Bing-Nan Su, Ren-Juan Shen, Zhuo-Lin Liu, Yang Li, Zi-Bing Jin

**Affiliations:** ^1^School of Basic Medical Sciences, Wenzhou Medical University, Wenzhou, China; ^2^Beijing Tongren Eye Center, Beijing Tongren Hospital, Beijing Institute of Ophthalmology, Capital Medical University, Beijing, China; ^3^Beijing Ophthalmology and Visual Sciences Key Laboratory, Beijing, China

**Keywords:** *USH2A*, mutation hotspot, Usher syndrome, retinitis pigmentosa, gene editing, inherited retinal dystrophy

## Abstract

**Purpose:**

Mutation in the *USH2A* gene is the most common cause of inherited retinal dystrophy (IRD), including non-syndromic retinitis pigmentosa (RP) and Usher syndrome II (USH2). Gene editing and therapy targeting *USH2A*, especially the hotspot region, would benefit a large proportion of IRD patients. In this study, we comprehensively analyzed the genetic spectrum of the *USH2A* gene, aiming to identify global hot spot mutations in *USH2A*-related IRDs and differences in hot spot regions across continents.

**Materials and methods:**

A retrospective *USH2A*-related IRD study was conducted, including our IRD cohort, and reported *USH2A* studies worldwide.

**Results:**

A total of 3,972 mutated *USH2A* alleles of approximately 1,935 patients were collected from 33 cohort studies worldwide, containing 102 alleles of 51 patients in our IRD cohort. Mutations in exon 13 were the most common, reaching 18.4% globally and a higher frequency of 22% in America, 19.2% in Europe, and a lower 12% in East Asia. Pathogenic mutations that affected 10 of the 72 exons of *USH2A*, exon 2, exon 13, exon 41–43, exon 50, exon 54, exon 57, exon 61, and exon 63 in total were responsible for half of global *USH2A* mutant alleles. With base editors including adenine base editor (ABE), cytidine base editor (CBE), and glycosylase base editor (GBE), 76.3% of single nucleotide variations (SNVs) and 58% of all mutations in *USH2A* are correctable. Meantime, four novel pathogenic mutations were revealed in our IRD cohort, p. (Val1130Cysfs*72), p. (Ala2139fs*14), p. (Gly4139Arg), and p. (Val4166Cysfs*7).

**Conclusion:**

In this study, we revealed four novel mutations, expanding the spectrum of *USH2A* mutations, and importantly presented global hotspot exons and mutations of *USH2A* as well as the proportion of SNVs that can be restored by different base editors, providing a perspective for exploring high-efficiency and broader-reaching gene editing and gene therapies.

## Introduction

Inherited retinal dystrophies (IRDs), affecting approximately one in 1,000 people worldwide, is a group of genetically and clinically heterogeneous disorders that severely destroy visual function. For many years, patients with IRDs had severe visual impairment and psychological burdens because lacking effective treatments. Retinitis pigmentosa (RP) is the most common form of IRD, affecting about 435,000 patients in China and almost two million worldwide (Berger et al., 2010).

Biallelic variants in the *USH2A* gene cause either Usher syndrome type 2 (USH2) or non-syndromic RP. In both conditions, the retinal phenotype includes progressive night blindness and constriction of the visual field, followed by central vision loss. In addition, USH2 is typically associated with congenital moderate to severe sensorineural hearing impairment (Weston et al., 2000; Hartong et al., 2006; Huang et al., 2015).

The *USH2A* gene was mapped to 1q41, spanning 800.05 kb with 72 exons, and encodes a transmembrane protein of 5202 amino acids named usherin, which contains laminin EGF motifs, a transmembrane domain, and repeats of fibronectin type III motifs. The usherin protein is predominantly expressed in the photoreceptor of the retina and the hair cells in the cochlea, and could be found in the basement membranes of many other tissues (Kimberling et al., 1995; Weston et al., 2000; van Wijk et al., 2004; Liu et al., 2007).

Many studies have shown that mutations in *USH2A* are responsible for at least 7% of non-syndromic RP and 57–67.7% of USH2. So far, more than 1,000 pathogenic or likely pathogenic variants have been reported in HGMD^1^ and ClinVar.^2^ Two ancestral variants, c.2299delG and c.2276G > T, are the most frequent in Europe and America (Carss et al., 2017; Stone et al., 2017). Two variants, c.2802T > G and c.8559-2A > G, are the most commonly reported in the Chinese cohort (Huang et al., 2015; Gao et al., 2021). The c.2299delG, c.2276G > T, and c.2802T > G are all located in exon 13, making exon 13 a hotspot region of *USH2A* mutations.

Research in gene editing and gene therapy of ocular disease has made rapid progress. Luxturna has been recently approved as a gene augmentation therapy drug for patients with *RPE65*-associated retinal dystrophy. Antisense oligonucleotide (AON) has also made impressive progress in treating IRDs. Three AON drugs have been used in clinical trials to treat IRDs (Slijkerman et al., 2016; Dulla et al., 2021). Base editing is one of the most recent and remarkable advances in gene editing. Base editors convert one base into another directly, without creating a double-strand DNA break (DSB), thereby minimizing the generation of excess undesired DSB-associated by-products. Up to now, three classes of DNA base editors have been established: ABE (converting A-T base pair to G-C base pair), CBE (converting C-G base pair to T-A base pair), and GBE (converting C-G base pair to G-C base pair) (Rees and Liu, 2018; Porto et al., 2020). SNVs make up most of the human pathogenic mutations, indicating that base editing will have great potential for treating a variety of genetic disorders (Goodwin et al., 2016; Landrum et al., 2016; Rees and Liu, 2018). Gene therapies and gene editing targeting *USH2A*, especially the hotspot mutant region, would benefit many patients with IRDs. In this study, we comprehensively analyzed the genetic spectrum of the *USH2A* gene, aiming to identify global hotspot mutant exons and variants in *USH2A*-related IRDs and differences in hotspot regions across continents. Furthermore, we predicted the proportion of *USH2A* mutations that could be corrected by base editing based on the mutation collection we integrated.

## Materials and methods

### Study subjects

The study was conducted in compliance with the tenets of the Declaration of Helsinki. Institutional Ethics approval and informed consent was obtained from the newly recruited patients in this study. Clinical and genetic diagnoses of patients in our IRD cohort of nearly 1,500 probands were reassessed. A medical history along with ophthalmic examinations was re-evaluated, including the initial symptoms, onset age, and best-corrected visual acuity (BCVA), fundus photography, optical coherence tomography (OCT), and full-field electroretinography (ERG). Pathogenicity of each variant was reassessed and categorized as pathogenic, likely pathogenic, uncertain significance, benign and likely benign according to ACMG/AMP classification guidelines (Richards et al., 2015). Variants categorized as benign or likely benign were excluded. Seventeen patients diagnosed with Usher syndrome and 34 patients with RP who were verified to harbor biallelic pathogenic mutations were enrolled. Informed consent was obtained from all study participants.

Global reported variants were collected through a comprehensive literature review. Keywords “*USH2A*”, “Usher”, “inherited retinal dystrophy”, and “retinitis pigmentosa” were used to search for genetic studies with a large sample size in the last 5–10 years. Genetic variation data were collected when available.

### Genetic test and classification of variants

Patients in our cohort underwent targeted exome sequencing (TES) or whole-exome sequencing (WES) were recruited. SNP array was used to detect the copy number variation (CNV) and real-time quantitative PCR was performed to confirm CNV. Suspected SNVs and small indels were verified by Sanger sequencing, as was the co-segregation analysis of available family members.

Multiple databases were used to annotate and evaluate the variants, and the pathogenicity of missense variants was predicted *in silico* by Mutation Taster, PolyPhen-2, and SIFT. The Human Gene Mutation Database and ClinVar database were screened for previously reported variants. Classification of variants was based on the ACMG guidelines (Richards et al., 2015).

### Genotype-phenotype correlation analysis

In total, 51 patients in our IRD cohort were classified into three categories based on different combinations of mutation types. Bilateral BCVA of patients was taken into analysis. To eliminate the inter-correlation of both eyes, linear mixed-effects model was used to explore the association between mutations and other variables (e.g., BCVA). Both Mean ± SD and median (interquartile range) were used for basic statistical description. Analysis was done using SAS 9.4. The significance level was set to be 0.05 and was adjusted according to Bonferroni criteria for multiple comparisons.

## Results

### Clinical manifestation in our inherited retinal dystrophy cohort

Fifty-one patients, including 17 USH and 34 RP, were included in the study after reassessment and identifying pathogenic *USH2A* biallelic mutations. All patients complained of night blindness and constriction of the visual field. Patients diagnosed with USH suffered different degrees of hearing loss. 64% of patients had onset of night blindness after 10 years old, consistent with previous reports that patients with *USH2A* mutations usually develop RP in the second decade of their life or later.

**FIGURE 1:**
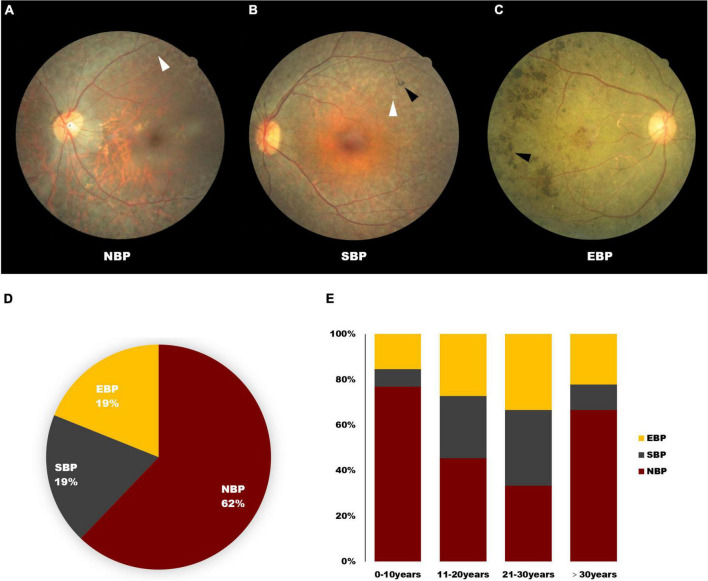
Clinical manifestations of *USH2A*-related inherited retinal dystrophy (IRD) patients. **(A–C)** Fundus photos present no bone-spicule pigmentation (NBP), scattered bone spicule (SBP), and extensive bone-spicule (EBP) in the posterior pole of the fundus. The black arrowheads indicate the bone-spicule pigmentation, white arrowheads indicated the RPE depigmentation presenting as small pale patches. **(D,E)** Distribution of *USH2A*-related IRDs patients presenting as NBP, SBP, and EBP. The X-axis of E represents the disease duration of <10, 11–20, 21–30, and >30 years.

All patients’ fundus showed typical RP presentations, including waxy pallor disk, attenuated retinal vasculature, and RPE degeneration. However, we also found a unique characteristic feature of RPE degeneration exhibited by *USH2A* mutated patients. In our cohort, patients were divided into three sub-groups, NBP (no bone-spicule), SBP (scattered bone spicule), and EBP (extensive bone-spicule) according to whether there was bone-spicule pigmentation in the posterior pole of the fundus (Figure 1). NBP was present in 62% of patients, and an additional 17% had sporadic bone-spicule deposits along the vascular arcade, which was defined as SBP (Figure 1D). Moreover, they all had the same presentation of RPE depigmentation, which manifests as small pale patches (Figure 1B).

Damage to photoreceptor and RPE function in patients with RP typically progresses from the periphery to the center. Interestingly, this NBP and SBP presentation did not strongly correlate with disease duration. However, among the four groups of different disease duration, the proportion of patients presenting NBP was the highest (Figure 1E).

### *USH2A* variants detected in our inherited retinal dystrophy cohort

**FIGURE 2:**
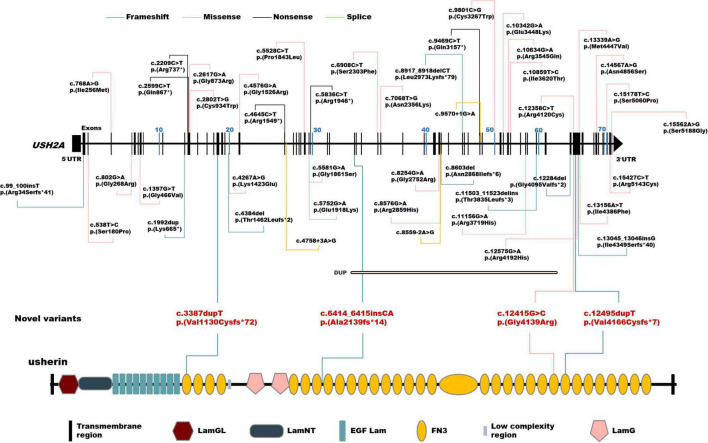
Schematic distribution of the *USH2A* variants identified in this study. Upper, schematic presentation of the *USH2A* gene and the distribution of variants identified in our inherited retinal dystrophy (IRD) cohort. Novel variants are highlighted in red. Lower, usherin domains encoded by *USH2A*. LamGL, laminin G-like domain; LamNT, laminin N-terminal domain; EGF_Lam, laminin-type epidermal growth factor-like domain; FN3, fibronectin type III domain; LamG, laminin G domain.

**FIGURE 3:**
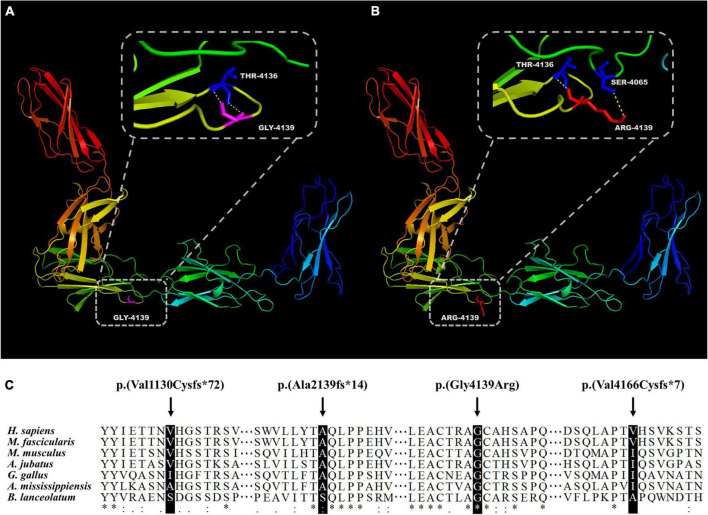
Evaluation of novel mutations in *USH2A*. Presentation of five adjacent FN3 domains and residue 4139 in wildtype **(A)** and mutant **(B)** usherin. **(C)** Alignment of the usherin protein sequence of residues 1130, 2139, 4139, and 4166 to its orthologous protein sequences in different species. The asterisk “*” indicates positions with a single and fully conserved residue, colon “:” indicates highly conserved positions, and “.” indicates positions with a weak conservation.

A total of 102 mutant alleles and 50 variants in *USH2A* were detected in our IRD cohort (Figure 2). Information of all *USH2A* variants in our IRD cohort was listed in Supplementary Table 1. Among the 102 alleles, 62 were missense mutations, and 40 were null mutations consisting of six stop-gain mutations, ten frameshift mutations, three splicing mutations, and a copy number variation. Four novel mutations, p. (Val1130Cysfs*72), p. (Ala2139fs*14), p. (Gly4139Arg), and p. (Val4166Cysfs*7) were newly reported, all of which were absent in the global healthy control database (Tables 1, 2). Despite valine at residue 1130 and 4166 being less conserved (Figure 3C), all three frameshift mutations will result in protein truncation, indicating strong evidence of pathogenicity. A patient diagnosed with typical non-syndromic RP harbored the novel missense mutation c.12415G > C, p. (Gly4139Arg). The p. (Gly4139Arg) mutation was located in an evolutionary conserved domain called fibronectin type III (FN3) and was predicted to be damaging or intolerant by multiple *in silico* predictive algorithms (Figure 2 and Table 2). A three-dimensional protein model of usherin was constructed using PyMOL to visualize the predicted impact of amino acid change at position 4139 (Figures 3A,B). As the model shows, the change from glycine to arginine would result in the formation of a new hydrogen bond between amino acid residues 4139 and 4065, which may affect the folding and stability of the protein. Co-segregation analysis in available family members showed the mutation segregated within the pedigree. Therefore, the p. (Gly4139Arg) mutation was defined as a likely pathogenic mutation according to the guideline of ACMG. The detailed clinical and genetic information of patients who harbored novel mutations was listed in Tables 1, 2.

**TABLE 1:** *USH2A* novel variants and clinical manifestation of these patients.

Patient ID	Diagnosis	Age at onset	Age at visit	BCVA (decimal)	Fundus (pigment)	Exon	Variant (NM_206933.2)	Protein change	References (PMID)
				**OD/OS**					
**R16**	RP	30	51	0.1/0.2	PB, SBP, and RPED	8	c.1397G > T	p. (Gly466Val)	24938718
						**33**	**c.6414_6415insCA**	p. (Ala2139fs*14)	**Novel**
**R17**	RP	16	42	NA	NA	**63**	**c.12495dupT**	p. (Val4166Cysfs*7)	**Novel**
						IVS48	c.9570 + 1G > A	Splice	23737954
**U12**	USH	20	45	OU HM/	PB, RPED (OD), and EBW (OS)	**17**	**c.3387dupT**	p. (Val1130Cysfs*72)	**Novel**
				30 cm		21	c.4576G > A	p. (Gly1526Arg)	25356976
**R22**	RP	30	45	0.3/0.2	PB and RPED	**63**	**c.12415G > C**	**p. (Gly4139Arg)**	**Novel**
						13	c.2802T > G	p. (Cys934Trp)	21686329

PB, peripheral bone-spicule; SBP, scattered bone spicule on the posterior pole; EBW, extensive bone-spicule throughout the whole retina; RPED, RPE depigmentation. Novel mutations are listed in bold.

**TABLE 2:** Pathogenicity of *USH2A* novel variants found in our cohort.

Variant	Protein change	Mutation taster	PolyPhen-2	SIFT	gnomAD AF (global)	ACMG
**c.3387dupT**	p. (Val1130Cysfs*72)	Disease-causing	/	/	Absent	P
**c.6414_6415insCA**	p. (Ala2139fs*14)	Disease-causing	/	/	Absent	P
**c.12495dupT**	p. (Val4166Cysfs*7)	Disease-causing	/	/	Absent	P
**c.12415G > C**	p. (Gly4139Arg)	Disease-causing	Probably damaging	Damaging	Absent	LP

P, pathogenic; LP, likely pathogenic.

### Genotype-phenotype correlation of our inherited retinal dystrophy cohort

**FIGURE 4:**
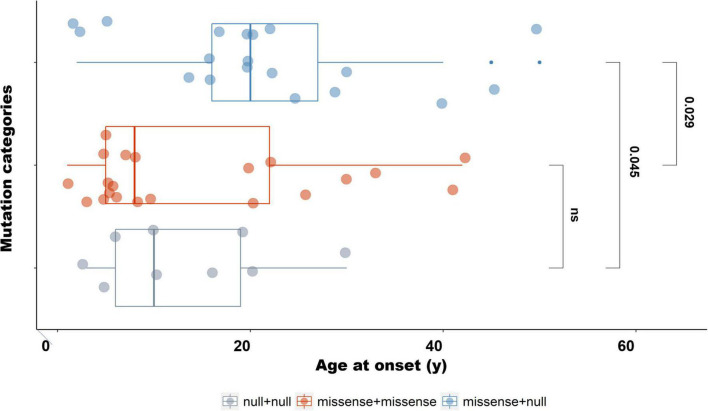
Age at onset of patients with different mutation types. The age at onset of patients with biallelic missense mutations was significantly younger than that of patients harboring one missense with one null mutation (P_*adjusted*_ = 0.029). The age at onset of patients with one missense and one null mutation was significantly older than that of patients with biallelic null mutations (P_*adjusted*_ = 0.045).

We classified the 50 different mutations detected in our IRD cohort into two categories: missense, and null (including nonsense, frameshift, splice, and copy number variation). The 51 patients were then divided into three groups based on different combinations of mutation types for two alleles: *USH2A^null/null^* (*n* = 10), *USH2A^missense/missense^* (*n* = 21), and *USH2A^null/missense^* (*n* = 20; Supplementary Table 3). Compared with patients with two null mutations, those patients who harbored two or one missense mutation were more likely to develop non-syndromic RP than USH (with an adjusted *P*-value of 0.040 and 0.019, respectively). There was no statistically significant difference between *USH2A^missense/missense^* and *USH2A^null/missense^* (Supplementary Table 4). Meanwhile, the age at onset of both patients with biallelic null mutations and patients with biallelic missense mutations was younger than that of patients with one missense and one null mutation (with an adjusted *P*-value of 0.045 and 0.029, respectively) (Figure 4 and Supplementary Table 5). There was no statistical difference in BCVA and disease progression among the three groups.

### The global spectrum of *USH2A* variants

A total of 3870 mutant *USH2A* alleles of approximately 1,884 patients were collected from 33 cohort studies with large sample sizes conducted in the United Kingdom, Spain, Germany, Italy, United States, Japan, Korea, China, etc. One hundred and two alleles of 51 patients detected in our IRD cohort were calculated together, making the sample size of 1,935 patients with 3972 mutant alleles (Supplementary Table 2).

**FIGURE 5:**
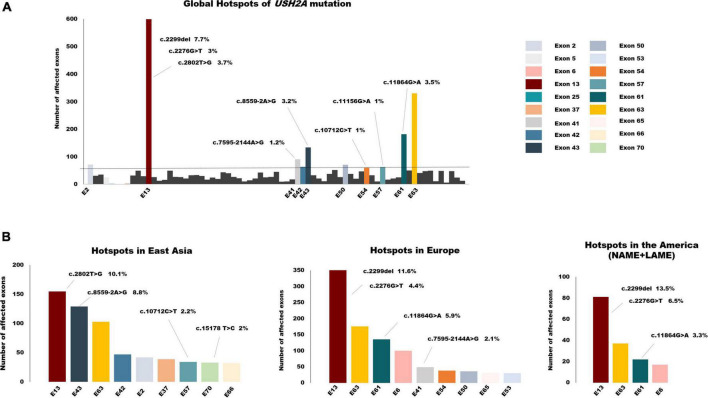
The global spectrum of *USH2A* variants. **(A)** Global hotspots of *USH2A* mutation. Exons colored are the top 10 hotspot mutant exons which accounted for more than half of all the alleles in total. **(B)** Hotspot exons and mutations of *USH2A* in different continents. Mutant alleles with a frequency of >1% were listed above the relevant exons.

Overall, the frequency of mutations detected in exon 13 was the highest, reaching 18.4%. The other two exons in the top3 were exon 63 with a frequency of 10% and exon 61 with 5.5%. Variants affected or detected in the following ten exons, including exon 2, exon 13, exon 41–43, exon 50, exon 54, exon 57, exon 61, and exon 63, accounting for more than half of the cohort. Global mutation hotspots are displayed in Figure 5A. Exon 13 harbored three of the top five hotspot variants: c.2299del, c.2802T > G, and c.2276G > T. The frameshift mutation c.2299del, p. (Glu767Serfs*21), which leads to the formation of truncated usherin protein, accounted for the highest frequency of 7.7% of *USH2A* mutations worldwide. Both missense mutations c.2802T > G, p. (Cys934Trp) and c.2276G > T, p. (Cys759Phe) were located in regions that encode the functional and evolutionary conserved EGF-Lam domain and were predicted to be detrimental to the correct folding of usherin (Xu et al., 2011; Reurink et al., 2022). Unexpectedly, no mutations occurred in exon 63 with a frequency higher than 1%, while the pathologic variants accounted for 3.5% of all the mutant alleles, ranking second.

Considering the small sample size of South Asia and Africa, we mainly focused on the mutation distribution in East Asia, Europe, and America (Figure 5B). As the mutation distributions in Latin America (LAME) and North America (NAME) were nearly identical, the data for these two regions were combined. There are some similarities among the three continents in the distribution of *USH2A* pathogenic variants. First, exon 13 was still the most commonly affected exon, with the highest frequency of 22% in America, 19.2% in Europe, and 12% in East Asia. Second, mutations that affected exon 63 frequently occurred in all three continents, with a nearly same frequency of around 10%. However, the mutation spectrum in different regions still varies, which may be due to the disparity in ethnicity. The splicing variant c.8559-2A > G, which has been demonstrated to result in skipping of exon 43 (Nakanishi et al., 2010), was common in the Asia cohort, especially in East Asia. Only one allele was reported in a study conducted in Germany. This splicing variant accounted for 8.8% of patients diagnosed with *USH2A*-related IRDs in East Asia, making exon 43 the second common mutant exon. Similarly, a deep intronic variant c.7595-2144A > G, leading to the insertion of an out-of-frame pseudo-exon (PE) (Vaché et al., 2012), was detected only in Caucasians and was one of the most frequent mutations in the Europe cohort.

### The strategy of base editing on *USH2A*

**FIGURE 6:**
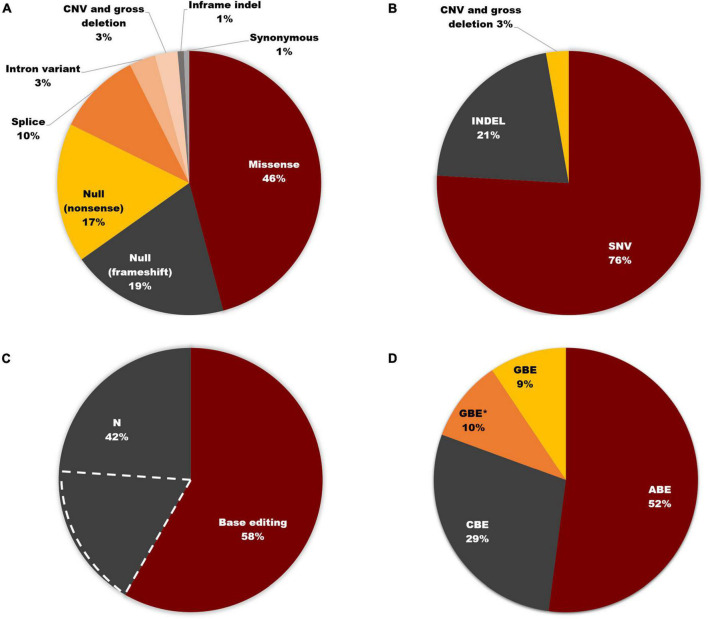
Base editing on *USH2A*. **(A)** Distribution of *USH2A* mutation types (*n* = 3,972 alleles). **(B)** SNVs account for 76% of all *USH2A* variants. **(C)** 58% of total variants can be corrected according to the editing principle of ABE, CBE, and GBE. The region marked by dashed white lines represents the SNVs that cannot be restored by three base editors. **(D)** Proportion of SNVs that can be corrected by different base editors. GBE with an asterisk (GBE*) represents SNVs in which the mutant nucleotide cannot be corrected through the original editing principle of ABE, CBE, and GBE, but the amino acid encoded will be restored by changing another base within the same codon using GBE.

Of the total 3972 mutant alleles listed in this study, missense variants comprised the highest proportion (46%). In contrast, null frameshift and nonsense variants, as well as splice site variants, accounted for 19, 17, and 10%, respectively (Figure 6A). In addition to causing missense mutations, point mutations can also lead to nonsense, splice site, and synonymous mutations. Therefore, as shown in Figure 6B, SNVs accounted for 76% of pathogenic *USH2A* variants, which is consistent with the consensus that SNVs are the most common mutation type in most human diseases (Goodwin et al., 2016; Landrum et al., 2016). 58% of total variants, which is 76.3% of SNVs, can be corrected according to the editing principle of ABE, CBE, and GBE. 23.7% of SNVs that involve alteration between purines and pyrimidines cannot be restored by these three base editors (Figure 6C). The proportion of SNVs that can be corrected by base editors was shown in Figure 6D. ABE made up the majority (52%), followed by CBE (29%) and GBE (19%). Notably, for a particular set of SNVs, the mutant nucleotide cannot be repaired according to the original editing principle of ABE (converting A-T base pair to G-C base pair), CBE (converting C-G base pair to T-A base pair), and GBE (converting G-C base pair to C-G base pair), but the amino acid encoded can be restored by changing another base within the same codon, thereby increasing the proportion of editable mutations. Excitingly, a hotspot mutation of *USH2A* in East Asia: c.2802T > G, p. (Cys934Trp) could be corrected by this strategy. Detailed information can be found in Supplementary Table 2.

## Discussion

Tremendous work has been done to seek therapeutic breakthroughs in hereditary retinal diseases (Chen et al., 2013; Mandai et al., 2017; Echigoya et al., 2018; Jin et al., 2019; Pendse et al., 2019; Zhang et al., 2021; Zhao et al., 2021). Significant progress has been achieved in research on cell replacement therapy, AON-based gene therapy, gene editing, etc. Among them, base editing has been promising to restore the vision in IRD patients. In the present study, we specifically described the possible application of three established base editors: ABE, CBE, and GBE on *USH2A*-related IRDs. Meanwhile, we predicted and delineated the proportion of *USH2A* mutations that can be corrected by base editing based on the global genetic landscape of *USH2A* that we depicted. SNV is the largest class of known human pathogenic mutations (Goodwin et al., 2016; Landrum et al., 2016). Thereby the correction of single point mutations will conceivably play an essential role in the future treatment of genetic disorders.

Among the *USH2A* SNVs, 18% of variants remained uncorrectable with these three base editors, and for all *USH2A* mutations, there are still 42% cannot be restored. The good news is that a new gene editing method termed “prime editing (PE)” has been developed. It is theoretically possible that PE can mediate indels and all SNVs (Anzalone et al., 2019), and a growing number of studies are underway to assess the editing efficacy, safety, and off-target concerns (Crunkhorn, 2021; Jiang et al., 2022).

Another promising approach for the future treatment of *USH2A*-related IRDs is exon skipping. Mutations in exon 13, including the recurrent mutations c.2299del, c.2276G > T, and c.2802T > G, are estimated to be responsible for approximately 18% of patients with *USH2A*-related IRDs around the world. Using antisense phosphorodiamidate morpholino oligomers (PMOs) to target exon 13 of zebrafish ush2a, Dulla et al. (2021) demonstrated that skipping of exon 13 using AON enables the synthesis of a shorter but functional USH2A protein, with the function of photoreceptor cells and retina restored.

Since mutations in *USH2A* are among the most common causes of non-syndromic RP and Usher syndromes, therapies targeting the hotspot mutant regions of *USH2A* would benefit a large proportion of IRD patients. In this large-scale retrospective study, we comprehensively reviewed nearly 4000 *USH2A* mutations from 33 genetic studies worldwide and presented the global mutation hotspot. Pathogenic mutations that affected ten of the 72 exons of *USH2A* were responsible for half of global *USH2A* mutant alleles. Thus, the rescue of dysfunction resulting from mutations on these exons may resolve more than half of *USH2A*-associated IRDs patients worldwide. Meanwhile, distribution and hotspot mutations of *USH2A* vary widely among different ethnic groups. For example, mutations in affected exon 43 were distinctly common in East Asia and the variant c.7595-2144A > G may be a unique mutation in Caucasians.

Although researchers from different countries have made a great effort to find genotype-phenotype correlations for *USH2A* mutations, no clear correlations have been established. It has been observed in a number of studies that patients with USH2 typically have an earlier onset and a faster clinical course of retinal degeneration than patients with non-syndromic RP. Protein-truncating variation has been demonstrated to correlate with earlier onset of vision loss (Blanco-Kelly et al., 2015; Lenassi et al., 2015; Pierrache et al., 2016). Furthermore, patients harboring biallelic truncating variants tended to exhibit more severe hearing loss than those with a single truncating variant or two non-truncating variants (Steele-Stallard et al., 2013). However, findings on genotype-phenotype correlation usually vary among studies of different scope and methodology. In our 51-patient cohort study, no statistical difference was found across three groups of different genotypes in disease progression and severity. Nevertheless, we found that patients harboring one or biallelic missense mutations were more likely to develop RP, which is similar to previous findings (Bork et al., 2001; Jiang et al., 2015; Inaba et al., 2020; Zhu et al., 2021). Meanwhile, the age at onset of both patients with biallelic null mutations and patients with both missense mutations was younger than that of patients with one missense and one null mutation, which is somewhat different from the findings of previous studies (Bork et al., 2001; Gao et al., 2021), indicating high heterogeneity of *USH2A*-related disorders. Patients with double null mutations presented a trend of reaching the state of low vision or legal blindness earlier than those who harbored one or no truncated mutations, but the differences were not statistically significant. Larger sample sizes and precise clinical follow-up information are needed. Clinical data of these large global samples need to be collected to search for a straightforward genotype-phenotype correlation.

In brief, we conducted a retrospective study of *USH2A*-related IRDs and provided a global mutation spectrum and hotspot in different regions, highlighting the critical therapeutic targets for the development of base editing treatments. Meanwhile, we revealed four novel mutations, expanding the spectrum of *USH2A* mutations, and reported a unique presentation of RPE depigmentation as patchy pale areas with no bone spicule on the posterior pole, increasing awareness of clinical manifestations of *USH2A*-related IRDs. Our findings provide a comprehensive perspective for exploring high-efficiency and broader-reaching gene editing and gene therapy.

## Data availability statement

The original contributions presented in this study are included in the article/Supplementary material, further inquiries can be directed to the corresponding author.

## Ethics statement

The studies involving human participants were reviewed and approved by Medical Ethics Committee of Beijing Tongren Hospital. The patients/participants provided their written informed consent to participate in this study.

## Author contributions

Z-BJ designed the study, recruited the patients, recorded clinical data, and revised the manuscript. R-JS and Z-LL performed the experiments, ophthalmic examinations, and genetic analysis. B-NS and R-JS reviewed the literature and performed the analysis. B-NS wrote the manuscript. YL performed data analysis and revised the manuscript. All authors contributed to the article and approved the submitted version.

## Footnotes

1
http://www.hgmd.cf.ac.uk/ac/all.php

2
www.ncbi.nlm.nih.gov/clinvar
